# Role of PPAR-δ in the development of zymosan-induced multiple organ failure: an experiment mice study

**DOI:** 10.1186/1476-9255-7-12

**Published:** 2010-02-18

**Authors:** Maria Galuppo, Rosanna Di Paola, Emanuela Mazzon, Tiziana Genovese, Concetta Crisafulli, Irene Paterniti, Elisabetta Cuzzocrea, Placido Bramanti, Amar Kapoor, Christoph Thiemermann, Salvatore Cuzzocrea

**Affiliations:** 1 Department of Clinical and Experimental Medicine and Pharmacology, School of Medicine, University of Messina, Italy; 2 IRCCS Centro Neurolesi "Bonino-Pulejo", Messina, Italy; 3 The William Harvey Research Institute, Centre for Experimental Medicine, Nephrology and Critical Care, St Bartholomew's, London, UK; 4 The Royal London School of Medicine and Dentistry, London, UK

## Abstract

**Background:**

Peroxisome proliferator-activated receptor (PPAR)-beta/delta is a nuclear receptor transcription factor that regulates gene expression in many important biological processes. It is expressed ubiquitously, especially white adipose tissue, heart, muscle, intestine, placenta and macrophages but many of its functions are unknown. Saturated and polyunsaturated fatty acids activate PPAR-beta/delta, but physiological ligands have not yet been identified. In the present study, we investigated the anti-inflammatory effects of PPAR-beta/delta activation, through the use of GW0742 (0,3 mg/kg 10% Dimethyl sulfoxide (DMSO) i.p), a synthetic high affinity ligand, on the development of zymosan-induced multiple organ failure (MOF).

**Methods:**

Multiple organ failure (MOF) was induced in mice by administration of zymosan (given at 500 mg/kg, i.p. as a suspension in saline). The control groups were treated with vehicle (0.25 ml/mouse saline), while the pharmacological treatment was the administration of GW0742 (0,3 mg/kg 10% DMSO i.p. 1 h and 6 h after zymosan administration). MOF and systemic inflammation in mice was assessed 18 hours after administration of zymosan.

**Results:**

Treatment with GW0742 caused a significant reduction of the peritoneal exudate formation and of the neutrophil infiltration caused by zymosan resulting in a reduction in myeloperoxidase activity. The PPAR-beta/delta agonist, GW0742, at the dose of 0,3 mg/kg in 10% DMSO, also attenuated the multiple organ dysfunction syndrome caused by zymosan. In pancreas, lung and gut, immunohistochemical analysis of some end points of the inflammatory response, such as inducible nitric oxide synthase (iNOS), nitrotyrosine, poly (ADP-ribose) (PAR), TNF- and IL-1as well as FasL, Bax, Bcl-2 and apoptosis, revealed positive staining in sections of tissue obtained from zymosan-injected mice. On the contrary, these parameters were markedly reduced in samples obtained from mice treated with GW0742

**Conclusions:**

In this study, we have shown that GW0742 attenuates the degree of zymosan-induced non-septic shock in mice.

## Background

Multiple organ dysfunction syndrome (MODS), previously known as multiple organ failure (MOF), is altered organ function in an acutely ill patient requiring medical intervention to achieve homeostasis. Patients suffering from multiple organ dysfunction syndrome comprise a heterogeneous population, which complicates research in its pathogenesis [1].

The condition usually results from infection, injury (accident, surgery), hypoperfusion and hypermetabolism. The primary cause triggers an uncontrolled local and systemic inflammatory response initiated by tissue damage. At present there is no agent that can reverse the established organ failure. Intraperitoneal injection of zymosan, in mice or rats leads, in the course of 1 to 2 weeks, to increasing organ damage and dysfunction [1].

The aim of this study is to show the therapeutic effect of GW0742, a PPAR β/δ treatment in mice zymosan-induced multiple organ failure.

## Introduction

Peroxisome proliferator-activated receptors (PPARs) are nuclear hormone receptors, i.e. ligand-dependent intracellular proteins that stimulate transcription of specific genes by binding to specific DNA sequences, following activation by an appropriate ligand. When activated, the transcription factors exert several functions in development and metabolism [2]. There are three PPAR subtypes encoded by separate genes, showing distinct but overlapping tissue distribution, and commonly designated as PPAR-α (NR1C1), PPAR-γ (NR1C3) and PPAR-β/δ (NUC1, NR1C2), or merely -δ [2,3]. In particular, PPAR-β/δ is an ubiquitous receptor, especially expressed in white adipose tissue, heart, muscle, intestine, placenta and macrophages [4]. It is activated by unsaturated or saturated long-chain fatty acids [5], prostacyclin, retinoic acid, and some eicosanoids [6]. Several animal studies reveal that PPAR-β/δ plays an important role in the metabolic adaptation of many tissues to environmental changes [2]. It appears to be implicated in the regulation of fatty acid metabolism of skeletal muscle and adipose tissue by controlling the expression of a gene involved in fatty acid uptake, β-oxidation and energy uncoupling [7-9].

In this study we wished to investigate the potential therapeutic role of PPAR-β/δ activation during an inflammatory process such as, multiple organ dysfunction syndrome (MODS, also known as multiple organ failure (MOF) or multiple organ system failure [10]) caused by zymosan. Multiple organ dysfunction syndrome is a cumulative sequence of progressive deterioration in function occurring in several organ systems, frequently seen after septic shock, multiple trauma, severe burns, or pancreatitis [11-13]. Zymosan is a non-bacterial, non-endotoxic agent derived from the cell wall of the yeast *Saccharomyces cerevisiae*. When injected into animals, it induces inflammation by inducing a wide range of inflammatory mediators [14-20]. It produces acute peritonitis and multiple organ failure characterized by functional and structural changes in liver, intestine, lung, and kidneys [16,18,21,22].

It is known that zymosan administration, in mice, within 18 h causes both signs of peritonitis and organ injury [23,24]. The onset of the inflammatory response caused by zymosan in the peritoneal cavity was associated with systemic hypotension, high peritoneal and plasma levels of NO, maximal cellular infiltration, exudate formation, cyclooxygenase activity and pro-inflammatory cytokines production [23,24]. In this model, we have studied the effect of GW0742, a synthetic high affinity ligand for PPAR-β/δ, after zymosan-induced injury.

## Materials and methods

### Animals

Male CD mice (20-22 g; Charles River; Milan; Italy) were housed in a controlled environment and provided with standard rodent chow and water. The study was approved by the University of Messina Review Board for the care of animals. Animal care was in compliance with Italian regulations on protection of animals used for experimental and other scientific purposes (D.M.116192) as well as with the EEC regulations (O.J. of E.C. L 358/1 12/18/1986)

### Zymosan-induced shock

Mice were randomly allocated into the following groups: (1) *Zymosan + vehicle group*. Mice were treated intraperitoneally (i.p.) with zymosan (500 mg/kg, suspended in saline solution, i.p.) and with the vehicle for GW0742 (10% dimethylsulfoxide (DMSO) (v/v) i.p), 1 and 6 h after zymosan administration, *n *= 10; (2) Zymosan + GW0742 *group*. Identical to the *Zymosan + vehicle group *but were administered GW0742 (0,3 mg/kg 10% DMSO i.p) at 1 and 6 hour after zymosan instead of vehicle, *n *= 10; (3) *Sham + vehicle group*. Identical to the *Zymosan + vehicle group*, except for the administration of saline instead of zymosan, *n *= 10; (4) *Sham + *GW0742 *group*. Identical to *Sham + vehicle group*, except for the administration of GW0742 (0,3 mg/kg in 10% DMSO i.p) 1 and 6 hour after saline administration, *n *= 10. Eighteen hours after administration of zymosan, animals were assessed for shock as described below. In another set of experiments, animals (*n *= 30 for each group) were randomly divided as described above and monitored for loss of body weight and mortality for 7 days after zymosan or saline administration.

### Clinical scoring of systemic toxicity

Clinical severity of systemic toxicity in the mice was scored during the experimental period, (7 days) after zymosan or saline injection, on a subjective scale ranging from 0 to 3; 0 = absence, 1 = mild, 2 = moderate, 3 = serious. The scale was used for each of the toxic signs (conjunctivitis, ruffled fur, diarrhea and lethargy) observed in the animals. The final score was produced upon totaling each evaluation (maximum value 12). All clinical score measurements were performed by an independent investigator, who had no knowledge of the treatment received by each respective animal.

### Assessment of acute peritonitis

Eighteen hours after zymosan or saline injection, all animals (n = 10 for each group) were killed under ether anesthesia in order to evaluate the development of acute inflammation in the peritoneum. Through an incision in the *linea alba*, 5 ml of phosphate buffered saline (PBS, composition in mM: NaCl 137, KCl 2.7, NaH_2_PO_4 _1.4, Na_2_HPO_4 _4.3, pH 7.4) was injected into the abdominal cavity. Washing buffer was removed with a plastic pipette and was transferred into a 10 ml centrifuge tube. The amount of exudate was calculated by subtracting the volume injected (5 ml) from the total volume recovered. Peritoneal exudate was centrifuged at 7000 × g for 10 min at room temperature.

### Peritoneal cell exudate collection and differential staining

At 18 h after treatment, the mice were anesthetized with intramuscular injection of ketamine/xylazine. The mice were injected with 5 mL of ice-cold RPMI-1640 medium (Gibco Inc., Grand Island, NY) with 10% heparin (50 U.I./ml), into the abdominal cavity. The peritoneal cavities were massaged for 1 min and the lavage fluid was collected. Peritoneal exudates cell (PEC) counts were carried out in a hemocytometer by mixing 100 μL of peritoneal cell exudate and 100 μL of eosin. The PEC was spin in a cytocentrifuge at 50 × g for 5 min onto a slide for the differential count. The slides were carefully removed and allowed to air dry briefly. PEC cytospins were stained with Wright-Giemsa stain. PEC cytospins were also stained with neutrophil/mast cell-specific chloroacetate esterase staining and macrophage/monocyte-specific alpha naphthyl butyrate esterase stains for the differential count.

### Measurement of nitrite/nitrate concentrations

Nitrite/nitrate (NO_2_/NO_3_) production, an indicator of NO synthesis, was measured in plasma and in the exudate samples collected 18 hours after zymosan or saline administration, as previously described [23,25]. Nitrate concentrations were calculated by comparison with OD550 of standard solutions of sodium nitrate prepared in saline solution.

### Immunohistochemical localization of nitrotyrosine, PARP, ICAM-1, P-Selectin, Bax, Bcl-2, TNF-α, IL-1β and FasL

Tyrosine nitration and PARP activation were detected, as previously described [26], in lung, liver and intestine sections using immunohistochemistry. At 18 hours after zymosan or saline injection, tissues were fixed in 10% (w/v) PBS-buffered formalin and 8 μm sections were prepared from paraffin embedded tissues. After deparaffinization, endogenous peroxidase was quenched with 0.3% (v/v) hydrogen peroxide in 60% (v/v) methanol for 30 min. The sections were permeabilized with 0.1% (v/v) Triton X-100 in PBS for 20 min. Non-specific adsorption was minimized by incubating the section in 2% (v/v) normal goat serum in PBS for 20 min. Endogenous biotin or avidin binding sites were blocked by sequential incubation for 15 min with avidin and biotin (Vector Laboratories, Burlingame, CA). The sections were then incubated overnight with 1:1000 dilution of primary anti-nitrotyrosine antibody (Millipore, 1:500 in PBS, v/v), anti-poly(ADP)-ribose (PAR) antibody (Santa Cruz Biotechnology, 1:500 in PBS, v/v), purified hamster anti-mouse ICAM-1 (CD54) (1:500 in PBS, w/v) (DBA, Milan, Italy), purified goat polyclonal antibody directed towards P-selectin which reacts with mice, anti-Bax rabbit polyclonal antibody (1:500 in PBS, v/v), anti-Bcl-2 polyclonal antibody rat (1:500 in PBS, v/v), anti-TNF-α antibody (Santa Cruz Biotechnology, 1:500 in PBS, v/v), anti-IL-1β antibody (Santa Cruz Biotechnology, 1:500 in PBS, v/v), or anti-Fas Ligand antibody (Abcam,1:500 in PBS, v/v). Controls included buffer alone or non-specific purified rabbit IgG. Specific labeling was detected with a biotin-conjugated specific secondary anti-IgG and avidin-biotin peroxidase complex (Vector Laboratories, Burlingame, CA). To verify the binding specificity for nitrotyrosine, PARP, ICAM-1, P-Selectin, Bax, Bcl-2, TNF-α and IL-1β and FasL, some sections were also incubated with primary antibody only (no secondary antibody) or with secondary antibody only (no primary antibody). In these situations, no positive staining was found in the sections indicating that the immunoreactions were positive in all the experiments carried out. In order to confirm that the immunoreactions for the nitrotyrosine were specific some sections were also incubated with the primary antibody (anti-nitrotyrosine) in the presence of excess nitrotyrosine (10 mM) to verify the binding specificity.

### Terminal deoxynucleotidyl transferase-mediated dUTP-biotin end labeling assay

Terminal deoxynucleotidyl transferase-mediated dUTP-biotin end labeling assay (TUNEL) was conducted by using a TUNEL detection kit according to the manufacturer's instruction (Apotag horseradish peroxidase kit; DBA, Milan, Italy). Briefly, sections were incubated with 15 2 g/Ml proteinase K for 15 min at room temperature and then washed with PBS. Endogenous peroxidase was inactivated by 3% H_2_O_2 _for 5 min at room temperature and then washed with PBS. Sections were immersed in terminal deoxynucleotidyl transferase (TdT) buffer containing deoxynucleotidyl transferase and biotinylated deoxyuridine 5-triphosphate in TdT buffer, incubated in a humid atmosphere at 37-C for 90 min, and then washed with PBS. The sections were incubated at room temperature for 30 min with anti-fluorescein isothiocyanate horseradish peroxidase-conjugated antibody, and the signals were visualized with diaminobenzidine.

### Subcellular fractionation, nuclear protein extraction and Western blot analysis for iNOS, IκB-α, NF-κB p65, Bax and Bcl-2

Tissues were homogenized in cold lysis buffer A (HEPES 10 mM pH = 7.9; KCl 10 mM;EDTA 0.1 mM; EGTA 0.1 mM; DTT 1 mM; PMSF 0.5 mM; Trypsin inhibitor 15 μg/ml; PepstatinA 3 μg/ml; Leupeptin 2 μg/ml; Benzamidina 40 μM). Homogenates were centrifuged at 12000 g for 3 min at 4°C, and the supernatant (cytosol + membrane extract) was collected to evaluate contents of iNOS, IkB-α, Bax, Bcl-2 and β-actin. The pellet was resuspended in buffer C (HEPES 20 mM; MgCl_2 _1.5 mM; NaCl 0.4 mM; EDTA 1 mM; EGTA 1 mM; DTT 1 mM; PMSF 0.5 μg/ml; Leupeptin 2 μg/ml; Benzamidina 40 μM; NONIDET P40 1%; Glicerolo 20%) and centrifuged at 12000 g for 12 min at 4°C, and the supernatant (nuclear extract) was collected to evaluate the content of NF-kB p65 and LaminB1. Protein concentration in the homogenate was determined by Bio-Rad Protein Assay (BioRad, Richmond CA) and 50 μg of cytosol and nuclear extract from each sample was analysed. Proteins were separated by 12% SDS-polyacrylamide gel electrophoresis and transferred on a PVDF membrane (Hybond-P Nitrocellulose, Amsherman Biosciences, UK). The membrane was blocked with 0.1% TBS-Tween containing 5% non fat milk for 1 h at room temperature. After the blocking, the membranes were incubated with the relative primary antibody overnight at 4°C; anti-iNOS TYPE II diluted 1:1000 (Transduction Laboratories), anti-IkB-α diluted 1:1000, anti-Bax diluted 1:500, anti-Bcl2 diluted 1:1000, anti-NFkB p65 diluted 1:250, anti-β-actin 1:5000 (Santa Cruz Biotechnology, CA) and anti-Laminin B1. After the incubation, the membranes were washed three times for ten minutes with 0.1% TBS Tween and were then incubated for one hour with peroxidase-conjugated anti- mouse or anti-rabbit secondary antibodies (Jackson ImmunoResearch Laboratories, USA) diluted 1:2000, the membranes were then washed three times for ten minutes and protein bands were detected with SuperSignal West Pico Chemioluminescent (PIERCE). Densitometric analysis was performed with a quantitative imaging system (ImageJ).

### Cytokines Production

The levels of TNF and IL-1β were evaluated in the plasma at 18 hours after zymosan or saline administration. The assay was conducted using a colorimetric commercial kit (Calbiochem-Novabiochem, La Jolla, CA). The ELISA has a lower detection limit of 10 pg/ml.

### Measurement of myeloperoxidase activity

Myeloperoxidase (MPO) activity, which was used as an indicator of PMN infiltration into the lung and intestinal tissues, was measured as previously described [27].

### Quantification of organ function and injury

Blood samples were taken at 18 h after zymosan or saline injection and centrifuged (1610 × *g *for 3 min at room temperature) to separate plasma. Levels of amylase, lipase, creatinine, alanine aminotransferase (ALT), aspartate aminotransferase (AST), bilirubine and alkaline phosphatase were measured by a veterinary clinical laboratory using standard laboratory techniques. For the evaluation of acid base balance and blood gas analysis (indicator of lung injury) arterial blood levels of pH, PaO_2 _and PaCO_2 _and bicarbonate ion (HCO_3_^-^) were determined by pH/Blood gases Analyser as previously described [28].

### Light microscopy

Lung, liver and small intestine samples were taken 18 hours after zymosan or saline injection. The tissue slices were fixed in Dietric solution [14.25% (v/v) ethanol, 1.85% (w/v) formaldehyde, 1% (v/v) acetic acid] for 1 week at room temperature, dehydrated by graded ethanol and embedded in Paraplast (Sherwood Medical, Mahwah, New Jersey, USA). Sections (thickness 7 μm) were deparaffinized with xylene, stained with hematoxylin and eosin and observed in Dialux 22 Leitz microscope.

### Materials

Unless stated otherwise, all reagents and compounds were obtained from Sigma Chemical Company (Milan, Italy).

### Data analysis

All values in the figures and text are expressed as mean ± standard error of the mean (s.e.m.) of *n *observations. For the *in vivo *studies, *n *represents the number of animals studied. In the experiments involving histology or immunohistochemistry, the figures shown are representative of at least three experiments (histological or immunohistochemistry coloration) performed on different experimental days on the tissue sections collected from all animals in each group. The results were analyzed by one-way ANOVA followed by a Bonferroni's *post-hoc *test for multiple comparisons. A *p*-value of less than 0.05 was considered significant. Statistical analysis for survival data was calculated by Kaplan-Meier survival analysis The Mann-Whitney U test (two-tailed, independent) was used to compare medians between the body weight and the clinical score. For such analyses, *p *< 0.05 was considered significant.

## Results

### Pancreas, lung and gut injury (histological evaluation) caused by zymosan is reduced in GW0742 treated mice

**Figure 1:**
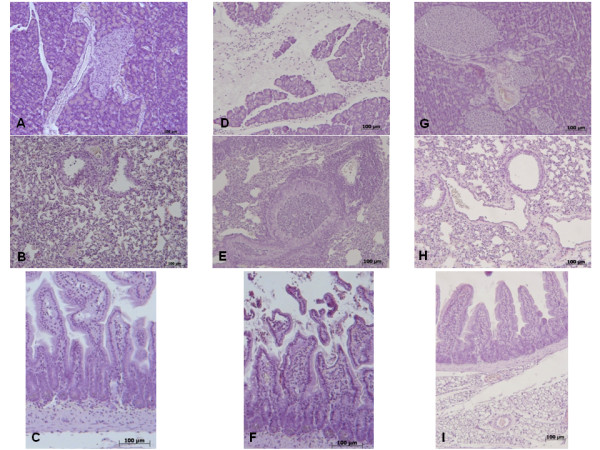
**No histological alteration was observed in the pancreas (a), lung (b) or gut (c) from sham-treated mice Pancreas (d) lung (e) and distal ileum (f) sections from zymosan-administered mice revealed morphological alterations and inflammatory cell infiltration**. Pancreas (**g**) lung (**h**) and distal ileum (**i**) from zymosan-administered mice treated with GW0742 demonstrated reduced morphological alterations and inflammatory cell infiltration. Figures are representative of at least 3 experiments performed on different experimental days.

At 18 h after zymosan administration, histological evaluation of pancreas (Figure 1D) lung (Figure 1E) and gut (Figure 1F) sections demonstrated several marked pathological changes. In the pancreas, there was extravasation of neutrophils (Figure 1D). Lung biopsy revealed inflammatory infiltration by neutrophils, macrophages and plasma cells (Figure 1E). In the gut, there was infiltration of inflammatory cells, edema in the space bounded by the villus, and separation of the epithelium from the basement membrane (Figure 1F). Treatment with GW0742 markedly reduced the histological damage in the pancreatic (Figure 1G), pulmonary (Figure 1H) and intestinal (Figure 1I) tissue. No histological alteration was observed in the pancreas (Figure 1A), lung (Figure 1B) or gut (Figure 1C) from sham-treated mice.

### Effect of GW0742-treatment on zymosan-induced body weight loss and mortality

**Figure 2:**
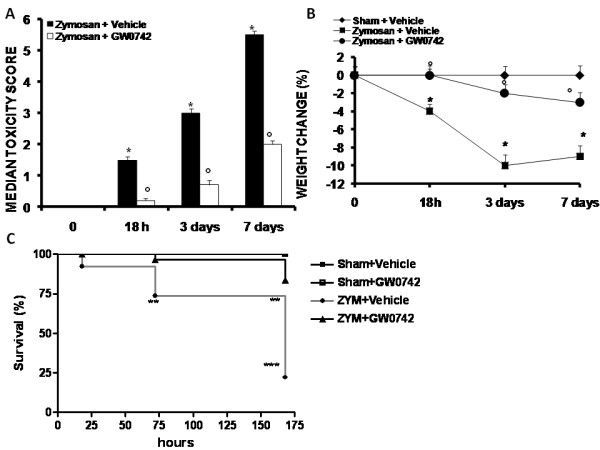
**Effect of GW0742-treatment on toxicity score (A), body weight change (B) and mortality (C)**. Data are means ± SEM of 10 mice for each group. *P < 0.01 *vs *sham, °P < 0.01 *vs *zymosan + vehicle.

Administration of zymosan caused severe illness in the mice, characterized by systemic toxicity and significant loss of body weight (Figure 2A, 1B). At the end of the observation period (7 days), 75% of zymosan-treated mice were dead (Figure 2C). Treatment with GW0742 reduced the development of systemic toxicity (Figure 2A), loss in body weight (Figure 2B) and mortality (Figure 2C), caused by zymosan. GW0742 treatment did not cause any significant changes in these parameters in sham mice (Figure 2A, 2B, 2C).

### Effect of GW0742 -treatment on inflammatory response in the peritoneal cavity

**Figure 3:**
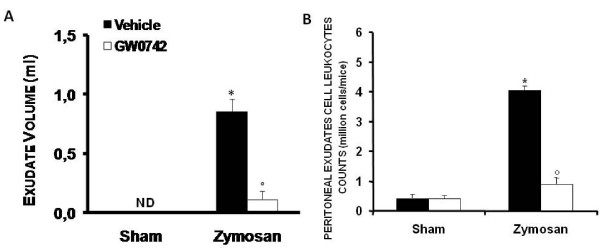
**Effect of GW0742-treatment on inflammatory response in the peritoneal cavity**. The increase in volume exudates (**A**) and peritoneal exudates cell leukocyte counts (**B**) in peritoneal cavity at 18 h after zymosan was reduced by GW0742 treatment. Data are mean ± standard deviation from n = 10 mice for each group. *P < 0.01 *vs *sham, °P < 0.01 *vs *zymosan + vehicle.

The development of acute peritonitis occurred 18 h after zymosan administration was indicated by the production of turbid exudates (Figure 3A). The total number of peritoneal exudate cells (PEC) (Figure 3B) was determined by trypan blue staining following intraperitoneal administration of zymosan or saline solution. This demonstrated a significant increase in the polymorphonuclear leukocyte number when compared with sham mice, which demonstrated no abnormalities in the peritoneal cavity or fluid.

Zymosan injection in mice was associated with an increase in PEC counts at 18 h, when compared to the saline controls (Figure 3B). Since there was a quantitative increase in PECs following zymosan injection, cytospin preparations were performed of the PEC for a differential estimation of the types of cells present. Wright-Giemsa stained slides of all controls appeared to contain mostly mononuclear cells including resident macrophages and lymphocytes and very few polymorphonuclear neutrophils, as previously demonstrated [29]. All cells appeared healthy and intact. At 18 h after zymosan administration, almost all cells appeared lysed, and because of excessive phagocytosis by the leukocytes, the neutrophils could not be differentiated from macrophages. Since the cells appeared lysed and the nucleus could not be differentiated, cell staining for specific esterases for neutrophil and macrophages were carried out in order to attempt differentiation between cell populations in the zymosan treated animals. In agreement with previous observations [29], we confirmed the presence of 90% mononuclear cells in the peritoneal cavity along with 10% PMNs in all the sham-treated animals. In contrast, the zymosan-treated samples could not be differentiated due to excessive phagocytosis and lysis of cells. Exudate formation (Figure 3A) and the degree of PEC count (Figure 3B) were significantly reduced in mice treated with GW0742.

### Effect of GW0742 on IκB-α degradation and NF-κB p65 activation

**Figure 4:**
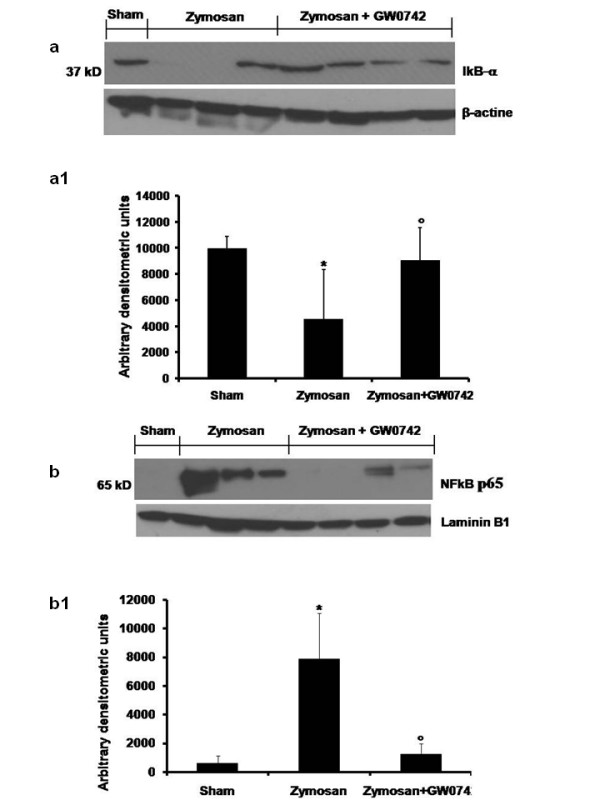
**Effect of GW0742-treatment on IkB-α degradation and NF-kB p-65 activation**. By Western Blot analysis, a basal level of IkB-α was detected in the lung tissue from sham-operated animals, whereas in zymosan-induced mice IkB-α levels were substantially reduced (**a**, see densitometric analysis **a1**). Treatment with GW0742 significantly increases the levels of IkB-α, after zymosan injection. Moreover, at 18 h following zymosan-treatment, the levels of NF-kB p-65 subunit protein in the nuclear fractions of the lung tissue were also significant increased compared to the sham-operated mice (**b**, see densitometric analysis **b1**). The levels of NF-kB p-65 protein were significantly reduced in the nuclear fractions of the lung tissues from animals that had received GW0742 treatment (**b**, see densitometric analysis **b1**). β-actin (**a**) and Laminin B1 (**b**) were used as internal control. The result in **a1 **and **b1 **are expressed as mean ± S.E. mean from five blots. P < 0.01 *vs *sham, °P < 0.01 *vs *zymosan + vehicle.

To investigate the inflammatory cellular mechanisms by which treatment with GW0742 may attenuate the development of zymosan-induced injury, we evaluated IκB-α degradation and nuclear NF-κB p65 translocation by Western Blot analysis. A basal level of IκB-α was detected in the lung tissues of sham-animals (Figure 4a, see densitometric analysis Figure 4a1), whereas in zymosan-treated mice, IκB-α levels were substantially reduced (Figure 4a, see densitometric analysis Figure 4a1). GW0742 prevented zymosan-induced IκB-α degradation, with IκB-α levels observed in these animals similar to those of the sham group (Figure 4a, see densitometric analysis Figure 4a1). In addition, zymosan administration caused a significant increase in NF-kB p65 levels in the nuclear fractions from lung tissues, compared to the sham-treated mice (Figure 4b, see densitometric analysis Figure 4b1). GW0742 treatment significantly reduced the levels of NF-kB p65 in the lung (Figure 4a, see densitometric analysis Figure 4a1).

### Effect of GW0742-treatment on cytokines production

**Figure 5:**
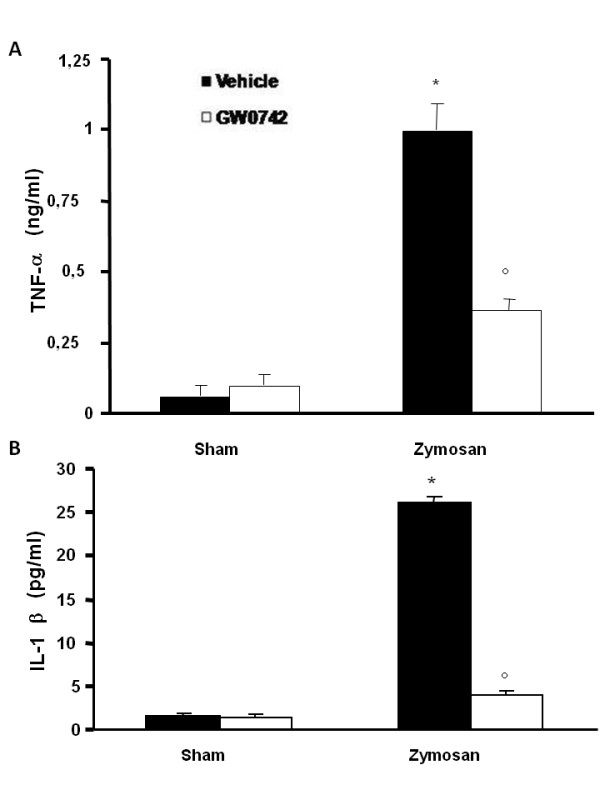
**Effect of GW0742 on plasma tumor necrosis factor alpha (TNF-α) and interleukin-1β (IL-1β) production A substantial increase in TNF-α (A) and IL-1β (B) production was found in tissues collected from zymosan-treated-mice compared to sham mice**. Plasma levels of TNF-α and IL-1β were significantly attenuated by the treatment with GW0742, 0.3 mg/Kg 10% DMSO i.p. at 1 and 6 hour after zymosan-injection (**A**, **B**, respectively). Data are mean ± standard deviation from n = 10 mice for each group. *P < 0.01 *vs *sham, °P < 0.01 *vs *zymosan + vehicle.

**Figure 6:**
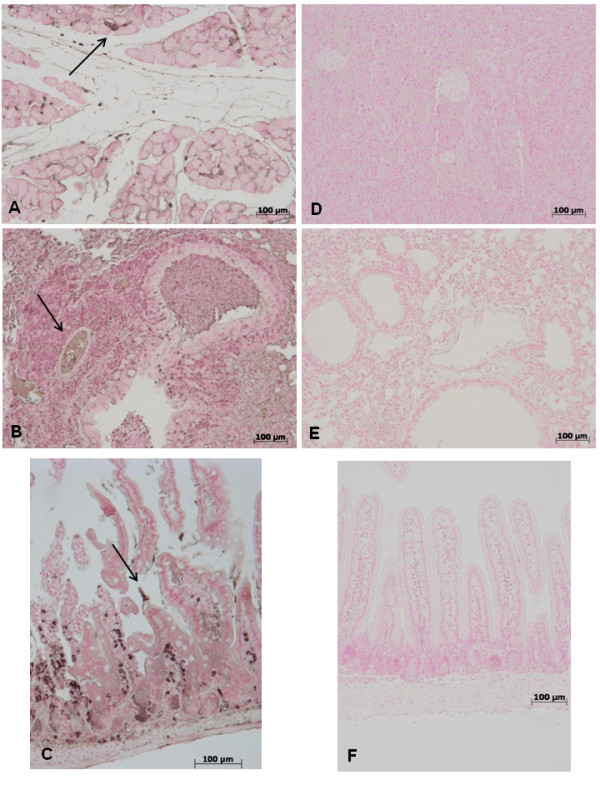
**and immunohistochemical localization of TNF-α in pancreas, lung and gut**. 18 hours following zymosan injection, a positive TNF-α staining was found in pancreas (A), lung (B) and gut (C). There was no detectable immunostaining for TNF-α in pancreas (**D**), lung (**E**) and gut (**F**) of zymosan-treated mice when mice were treated with GW0742. Figures are representative of at least 3 experiments performed on different experimental days.

The modulation of GW0742 on the inflammatory process through the regulation of cytokine secretion was assessed by determination of plasmatic levels of the pro-inflammatory cytokines TNF-α and IL-1β. A substantial increase in TNF-α and IL-1β formation was observed in zymosan-treated mice when compared to sham mice (Figure 5A, 5B, respectively), while a significant inhibition of TNF-α and IL-1β was observed when animals with zymosan-induced injury were treated with GW0742 (Figure 5B, respectively). In addition, tissue sections of pancreas, lung and gut obtained from animals 18 h after zymosan administration, demonstrated positive staining for TNF-α and IL-1-β in pancreas, (Figure 6A, 7A respectively) lung (Figure 6B, 7B respectively) and gut(Figure 6C, 7C respectively) On the contrary the staining for TNF-α and IL-1-β was visibly and significantly reduced in zymosan mice treated with GW0742 in pancreas, (Figure 6D, 7D respectively) lung (Figure 6E, 7E respectively) and gut (Figure 6F, 7F respectively). In the pancreas, lungs and gut of sham animals no positive staining was observed for TNF-α (data not shown) or IL-1-β (data not shown).

**Figure 7:**
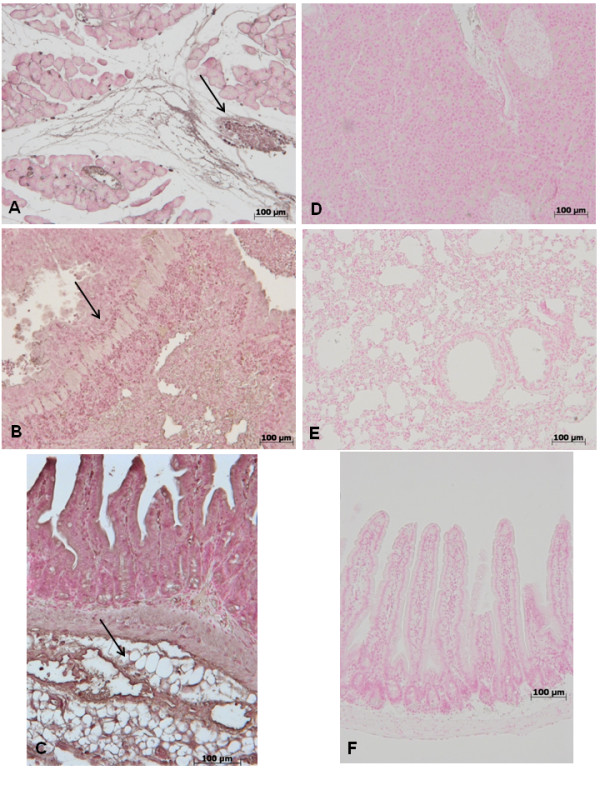
**immunohistochemical localization of IL-1β in pancreas, lung and gut**. 18 hours following zymosan injection, a positive IL-1β staining was found in pancreas (A), lung (B) and gut (C). There was no detectable immunostaining for IL-1β in pancreas (**D**), lung (**E**) and gut (**F**) of zymosan-treated mice when mice were treated with GW0742. Figures are representative of at least 3 experiments performed on different experimental days.

### Effect of GW0742- treatment on ICAM and P-selectin expression

**Figure 8:**
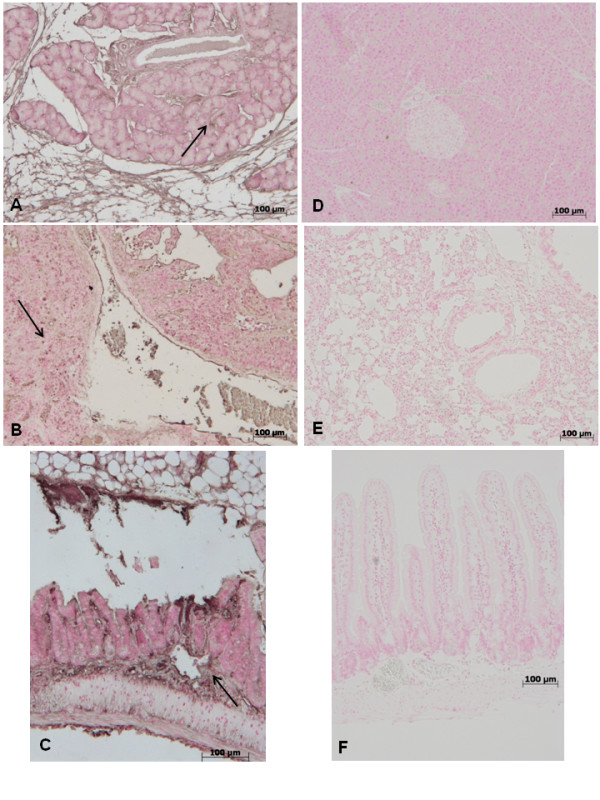
**Immunohistochemical localization of ICAM-1 in pancreas, lung and gut**. 18 hours following zymosan injection, a positive ICAM-1 staining was found in pancreas (**A**), lung (**B**) and gut (**C**). There was no detectable immunostaining for ICAM-1 in pancreas (**D**), lung (**E**) and gut (**F**) of zymosan-treated mice when mice were treated with GW0742. Figures are representative of at least 3 experiments performed on different experimental days.

At 18 h after zymosan administration, expression of the adhesion molecules ICAM-1 and P-selectin were evaluated to assess neutrophil infiltration. In zymosan-treated mice, an increase of immunohistochemical staining for ICAM-1 and P-selectin was demonstrated in the pancreas (Figure 8A, 9A respectively) lung (Figure 8B, 9B respectively) and gut(Figure 8C, 9C respectively), (see arrows) while the immunostainings for ICAM-1 and P-selectin were markedly reduced in pancreas (Figure 8D, 9D respectively) lung (Figure 8E, 9E respectively) and gut(Figure 8F, 9F respectively) tissues obtained from mice, that were treated with GW0742. No staining for either ICAM-1 or P-selectin was found in tissue sections obtained from sham-treated mice (data not shown).

**Figure 9:**
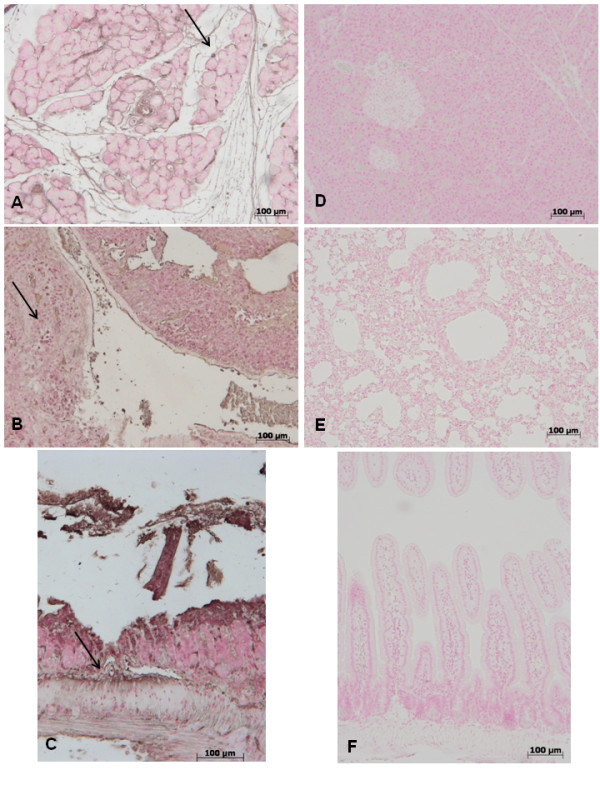
**Immunohistochemical localization of P-Selectin in pancreas, lung and gut**. 18 hours following zymosan injection, a positive P-Selectin staining was found in pancreas (**A**), lung (**B**) and gut (**C**). There was no detectable immunostaining for P-Selectin in pancreas (**D**), lung (**E**) and gut (**F**) of zymosan-treated mice when mice were treated with GW0742. Figures are representative of at least 3 experiments performed on different experimental days.

### Effect of GW0742- treatment on inflammatory cell infiltration

**Figure 10:**
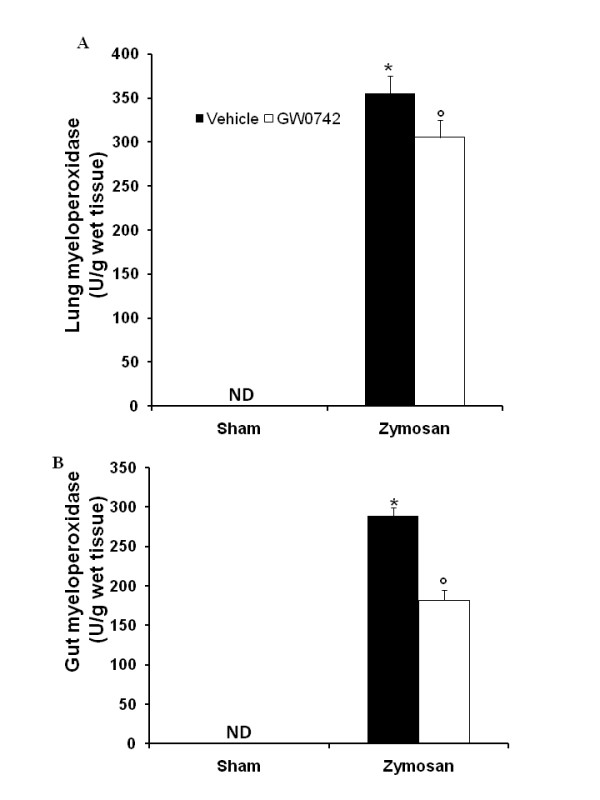
**Moreover, MPO activity in lung (A) and gut (B) samples of zymosan-treated-mice was significantly increased in comparision to sham mice (A and B, respectively)**. Treatment with GW0742 significantly reduced the increase of MPO activity in lung (**A**) and gut (**B**). Data are mean ± standard deviation from n = 10 mice for each group. *P < 0.01 *vs *sham, °P < 0.01 *vs *zymosan + vehicle.

The accumulation of neutrophils in the intestine and lung is a hallmark of multiple organ failure induced by zymosan. An indirect assessment of neutrophil infiltration was carried out by measuring the activity of myeloperoxidase (MPO), an enzyme that is contained in (and specific for) PMN lysosome dysfunction [23,25]. At 18 h after zymosan administration, MPO activity was significantly increased in the lungs (Figure 10A) and gut (Figure 10B) of zymosan-challenged mice, when compared with sham-operated mice (Figure 10A, B). MPO activity was markedly reduced in the lungs (Figure 10A) and gut (Figure 10B) of zymosan-challenged mice treated with GW0742.

### Effect of GW0742 treatment on NO formation and iNOS expression

**Figure 11:**
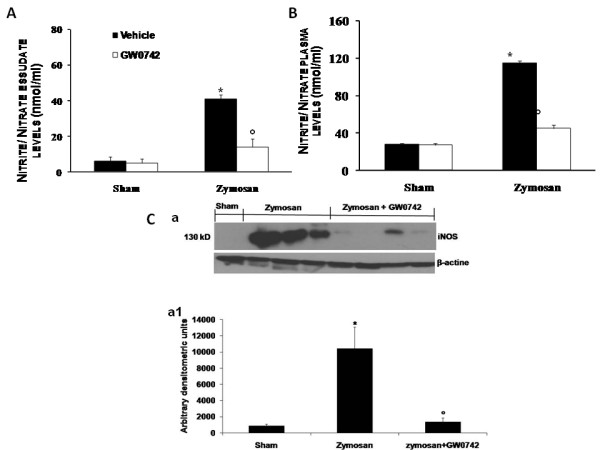
**Effect of GW0742 on peritoneal exudates (A) and plasma nitrate/nitrite levels (B)**. Nitrate/nitrite levels were significantly increased both in peritoneal exudate and in plasma of zymosan-treated mice in comparison to vehicle group (sham group). GW0742 reduced the zymosan-induced increase of nitrate/nitrite levels in peritoneal exudate **(A) **and in plasma **(B)**. In addition, by Western Blot analysis the iNOS expression was evaluated in the lung. At 18 h after zymosan administration a significant increase in the iNOS expression was observed in the ileum (**Ca**, see densitometric analysis **a1**) compared to the sham-treated mice. GW0742 treatment significantly reduced the iNOS expression (**Ca**, see densitometric analysis **a1**). The intensity of bands was measured using a phosphoimager in all the experimental groups. β-actin was used as internal control. A representative blot of lysates obtained from each group is shown, and densitometric analysis of all animals is reported (n = 5 mice from each group). Data are mean ± standard deviation from n = 10 mice for each group. *P < 0.01 *vs *sham, °P < 0.01 *vs *zymosan + vehicle.

The analysis of exudates (Figure 11A) and plasma (Figure 11B) levels validate the biochemical and inflammatory changes observed in the peritoneal cavity of zymosan-treated mice, showing a significant increase of nitrite/nitrate (NOx) concentration (Figure 11A, 11B, respectively) when compared with sham-operated mice. These values were significantly reduced in mice treated with GW0742 (Figure 11A, 11B, respectively). We assessed iNOS expression in samples of pulmonary tissue by Western Blot analysis (Figure 11Ca, see densitometric analysis a1). A significant increase in iNOS expression has been demonstrated in samples of lung obtained from zymosan-injected mice, when compared with sham-operated mice (Figure 11Ca, see densitometric analysis a1). In contrast, a significant decrease in iNOS expression was clearly observed in the zymosan-challenged mice treated with GW0742 (Figure 11Ca, see densitometric analysis a1).

### Effect of GW0742 -treatment on nitrosative stress and PARP activation

**Figure 12:**
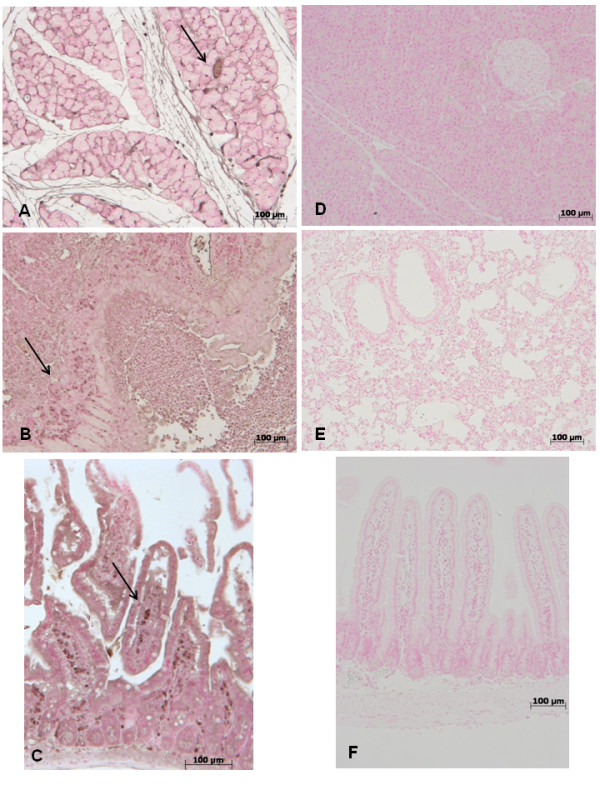
**Immunohistochemical localization of nitrotyrosine in pancreas, lung and gut**. 18 h following zymosan injection, a positive nitrotyrosine staining was found in the pancreas (**A)**, lung (**B**) and gut (**C**). There was no detectable immunostaining for nitrotyrosine in the pancreas (**D**), lung (**E**) and gut (**F**) of zymosan-administered mice when mice were treated with GW0742. Figure is representative of at least 3 experiments performed on different experimental days.

To determine the localization of *"peroxynitrite formation" *and/or other nitrogen derivatives produced during multiple organ failure, zymosan-induced nitrotyrosine, a specific marker of nitrosative stress, was measured by immunohistochemical analysis in sections of pancreas, lung and gut tissues, using a specific anti-nitrotyrosine antibody. The samples obtained from sham-operated mice did not stain for nitrotyrosine (data not shown), while sections from zymosan-induced mice exhibited positive staining for nitrotyrosine in pancreas (Figure 12A), lung (Figure 12B) and gut (Figure 12C) tissues (see arrows). A marked reduction in nitrotyrosine staining was found in the pancreas (Figure 12D), lung (Figure 12E) and gut (Figure 12F) of the zymosan-challenged mice treated with GW0742.

**Figure 13:**
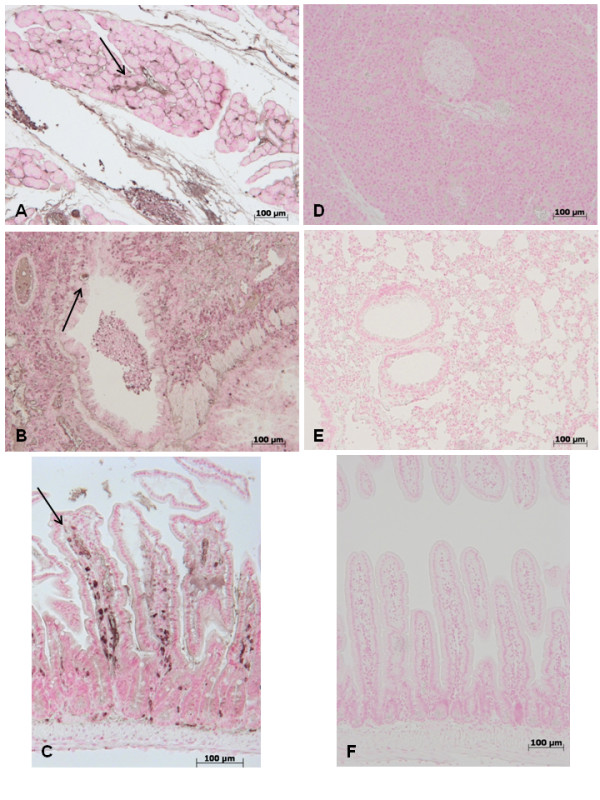
**Immunohistochemical localization of PAR in pancreas, lung and gut**. 18 h following zymosan injection, a positive PAR staining was found in the pancreas (**A**), lung (**B**) and gut (**C**). There was no detectable immunostaining for PAR in the pancreas (**D**), lung (**E**) and gut (**F**) of zymosan-administered mice when mice were treated with GW0742. Figure is representative of at least 3 experiments performed on different experimental days.

Sections of pancreas, lung and gut were taken at 18 h after zymosan administration in order to determine the activation of the nuclear enzyme, poly (ADP-ribose) polymerase (PARP), that has been implicated in the pathogenesis of multiple organ failure. Thus, we used an immunohistochemical approach to assess the presence of PAR, an indicator of PARP activation *in vivo*. There was positive staining for PAR localized in sections of pancreas (Figure 13A), lung (Figure 13B) and gut (Figure 13C) obtained from zymosan-challenged mice. Treatment with GW0742 reduced the degree of positive staining for PAR in the pancreas (Figure 13D), lung (Figure 13E) and gut (Figure 13F). No positive staining for PAR was identified in tissues from Sham-operated mice (data not shown).

### Effect of GW 0742 -treatment on Fas-ligand expression and apoptosis

**Figure 14:**
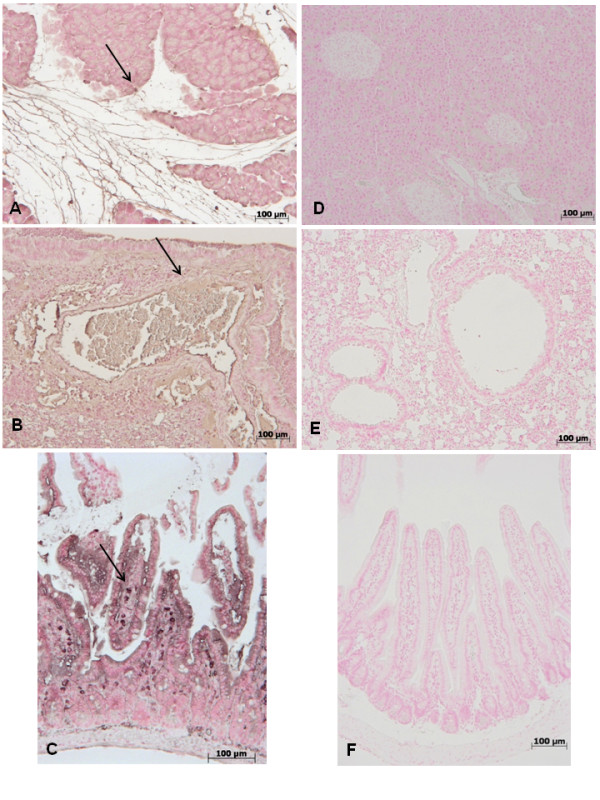
**Effect of GW0742 on Fas-ligand expression**. 18 h following zymosan injection, positive FasL staining was found in pancreas (**A**), lung (**B**) and gut (**C**). There was no detectable immunostaining for FasL in pancreas (**D**), lung (**E**) and gut (**F**) of zymosan-administered mice when mice were treated with GW0742. Figures are representative of at least 3 experiments performed on different experimental days.

Immunohistological staining for the Fas Ligand in the pancreas (Figure 14A), lung (Figure 14B) and gut (Figure 14C) were determined 18 h after zymosan-induced injury. Tissue sections from the sham-operated mice did not stain for the Fas Ligand (data not shown), whereas sections obtained from the zymosan-challenged mice exhibited positive staining for the Fas Ligand, in the pancreas (Figures 14A), lung (Figure 14B) and gut (Figure 14C). Treatment with GW0742 reduced the degree of positive staining for the Fas Ligand in the pancreas (Figures 14D), lung (Figure 14E) and gut (Figure 14F).

**Figure 15:**
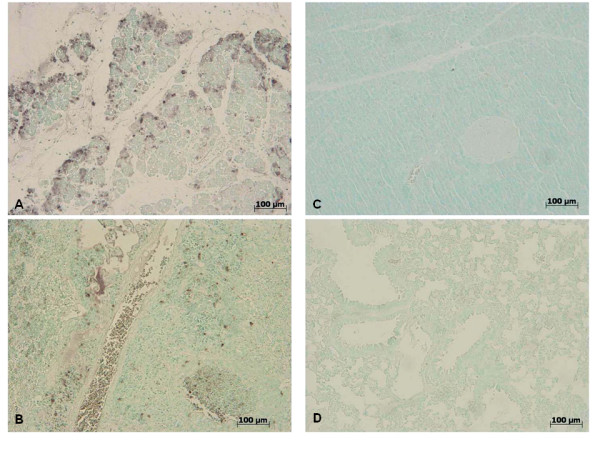
**Effect of GW0742 on TUNEL-like staining**. At 18 h after zymosan administration, TUNEL-like staining showed a marked appearance of dark brown apoptotic cells and intercellular apoptotic fragments in pancreatic (**A**) pulmonary (**B**) tissue. On the contrary, the number of dark brown cells was significantly reduced in pancreas (**C**) and lung (**D**) by the treatment with GW0742. Figures are representative of at least 3 experiments performed on different experimental days.

To test whether tissue damage was associated with cell death by apoptosis, we assessed TUNEL-like staining in pancreas (Figure 15A) and pulmonary tissue (Figure 15B). Almost no apoptotic cells were detectable in sections of pancreatic and pulmonary tissue in sham-operated mice (data not shown). At 18 h after zymosan-induced injury, sections of pancreas (Figure 15A) and lung (Figure 15B) demonstrated a marked appearance of dark brown apoptotic cells and intercellular apoptotic fragments. In contrast, pancreatic (Figure 15C) and pulmonary (Figure 15D) tissue obtained from zymosan-administered mice treated with GW0742, demonstrated a small number of apoptotic cells or fragments.

### Western blot analysis and immunohistochemistry for Bax and Bcl-2

**Figure 16:**
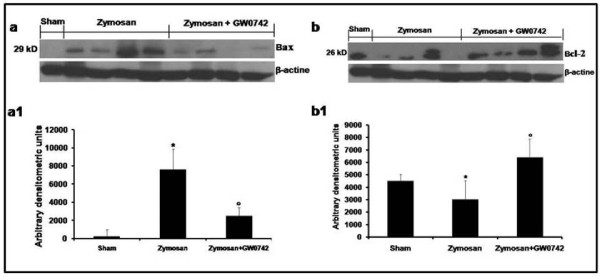
**Western Blot analysis of Bax and Bcl-2 expression**. Representative Western Blot of Bax and Bcl-2 levels was realized in pulmonary samples at 18 h after zymosan-injection. At 18 h after zymosan administration, a significant increase in the Bax expression was observed in the lung (**A**, see densitometric analysis **A1**) compared to the sham-treated mice, whereas in GW0742-treated mice Bax levels were substantially reduce (A, see densitometric analysis **A1**). On the contrary, at 18 h after zymosan administration, a decrease in the Bcl-2 expression was observed in the lung (**B**, see densitometric analysis **B1**) compared to the sham-treated mice, while Bcl-2 expression was more evident in the pulmonary tissue from zymosan-treated mice that received GW0742 treatment (**B**, see densitometric analysis **B**). Data are mean ± standard deviation from n = 10 mice for each group. *P < 0.01 *vs *sham, °P < 0.01 *vs *zymosan + vehicle.

The presence of Bax and Bcl-2 in lung homogenates was investigated by Western blot 18 hours after zymosan administration. A basal level of Bax was detected in lung tissues obtained from sham-treated animals (Figure 16A, see densitometric analysis Figure 16A1). Bax levels were substantially increased in the lung tissues from zymosan-administered mice (Figure 16A, see densitometric analysis Figure 16A1). In contrast, GW0742 treatment prevented the zymosan-induced Bax expression (Figure 16A, see densitometric analysis Figure 16A1). A basal level of Bcl-2 was detected in lung tissues obtained from sham-treated animals (Figure 16B, see densitometric analysis Figure 16B1). Bcl-2 levels were substantially reduced in the lung tissues from zymosan-administered mice (Figure 16B, see densitometric analysis Figure 16B1). In contrast, GW0742 treatment significantly attenuated the zymosan-induced reduction of Bcl-2 expression (Figure 16B, see densitometric analysis Figure 16B1).

**Figure 17:**
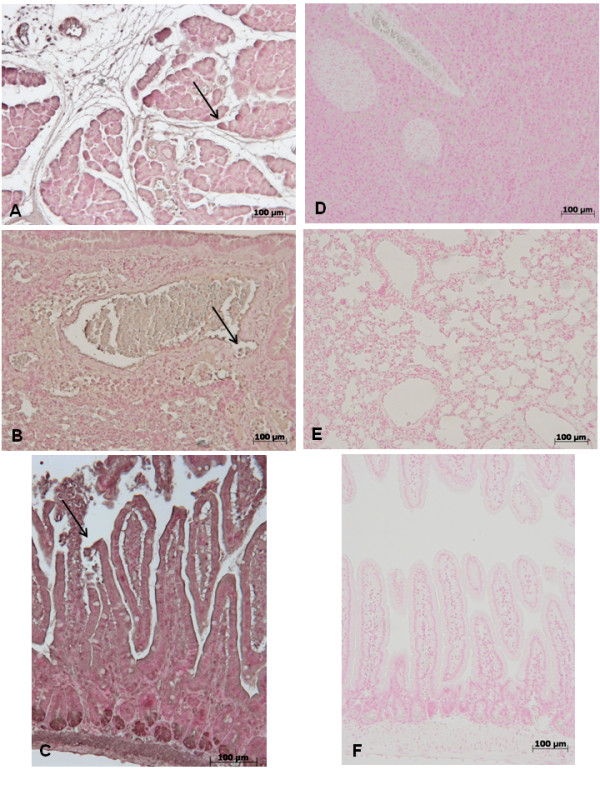
**Immunohistochemical localization of Bax**. Moreover, Bax expression was also evaluated by immunohistochemical analysis in pancreas, lung and gut. 18 hours following zymosan injection, positive Bax staining was found in pancreas (**A**), lung (**B**) and gut (**C**). No positive staining for Bax was detected in pancreas (**D**), lung (**E**) and gut (**F**) of zymosan-treated mice when mice were treated with GW0742. Figures are representative of at least experiments performed on different experimental days.

**Figure 18:**
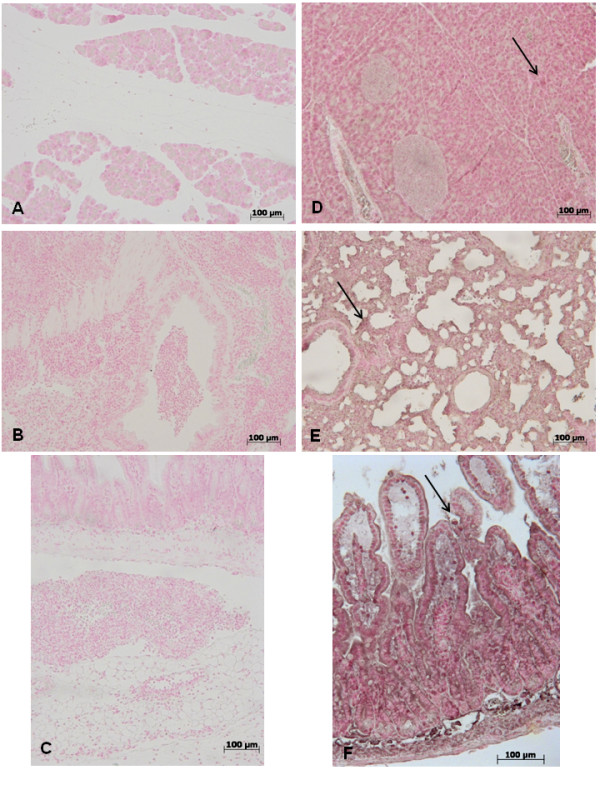
**Immunohistochemical localization of Bcl-2 expression**. Moreover, Bcl-2 expression was also evaluated by immunohistochemical analysis in pancreas, lung and gut. At 18 h after zymosan administration, no positive staining for Bcl-2 was observed in pancreas (**A**), lung (**B**) and gut (**C**) from zymosan-treated mice. On the contrary, positive staining for Bcl-2 was observed in pancreas (**D**), lung (**E**) and gut (**F**) from mice treated with GW0742. Figures are representative of at least experiments performed on different experimental days.

To determine the immunohistological staining for Bax (Figure 17) and Bcl-2 (Figure 18), samples of pancreas (Figure 17A, 18A respectively) lung (Figure 17B, 18B, respectively) and gut (Figure 17C, 18C respectively) were also collected 18 hours after zymosan administration. Tissues taken from sham-treated mice did not stain for Bax (data not shown), whereas pancreatic (Figure 17A), pulmonary (Figure 17B) and intestinal (Figure 17C) sections obtained from zymosan-treated mice exhibited positive staining for Bax. GW0742 treatment reduced the degree of positive staining for Bax in the pancreas (Figure 17D), lung (Figure 17E) and gut (Figure 17F) of mice subjected to zymosan-induced injury.

In addition, lung sections from sham-treated mice demonstrated positive staining for Bcl-2 (data not shown), whereas in zymosan-administered mice, Bcl-2 staining was significantly reduced (Figure 18). GW0742 treatment significantly attenuated the loss of positive staining for Bcl-2 in pancreatic (Figure 18D), pulmonary (Figure 18E) and intestinal (Figure 18F) samples of mice subjected to zymosan-induced injury.

### Zymosan-induced multiple organ dysfunction syndrome is reduced by GW0742

#### Effects on lung injury

**Figure 19:**
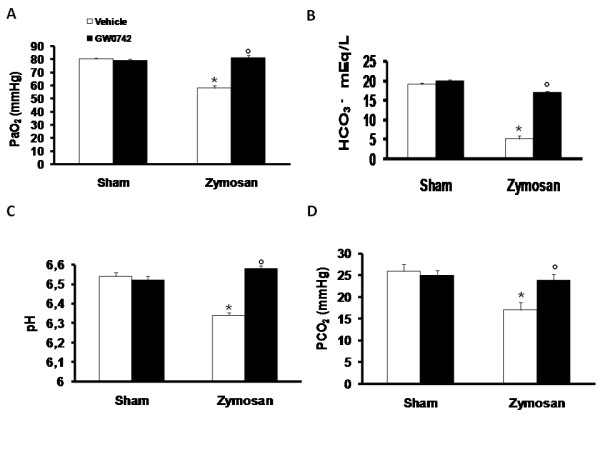
**Effect of GW0742 on lung injury**. Zymosan administration resulted in significant fall in the arterial levels of PaO_2 _(**A**), PCO_2 _(**B**), pH (**C**) and HCO_3_^-^(**D**). Administration of GW0742 prevents the lung dysfunctions. Data are means ± SEM of 10 mice for each group. *P < 0.01 *vs *sham, °P < 0.01 *vs *zymosan + vehicle.

When compared to sham-operated mice, zymosan-administered mice demonstrated significant alterations in the PaO_2 _(Figure 19A), PCO_2 _(Figure 19B), HCO_3_^-^(Figure 19C) and pH arterial blood levels (Figure 19D), suggesting the development of lung dysfunction. In contrast, treatment with GW0742 significantly reduced the lung injury caused by zymosan (Figure 19A, 19B, 19C, 19D).

#### Hepatocellular injury

**Figure 20:**
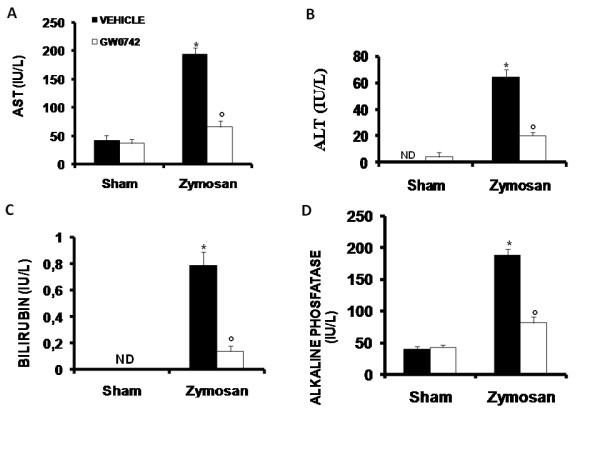
**Effect of GW0742 on liver injury**. Administration of zymosan resulted in significantly increased levels of plasma AST (**A**), ALT (**B**), bilirubin (**C**) and alkaline phosphatase (**D**). GW0742 treatment significantly reduced all these parameters in zymosan treated mice. Data are mean ± standard deviation from n = 10 mice for each group. *P < 0.01 *vs *sham, °P < 0.01 *vs *zymosan + vehicle.

When compared to sham-operated mice, zymosan-administered mice demonstrated significantly high plasma concentrations of AST (Figure 20A), ALT (Figure 20B), bilirubin (Figure 20C) and alkaline phosphatase (Figure 20D), suggesting the presence of a consistent hepatocellular injury. In contrast, treatment with GW0742 significantly reduced the liver injury caused by zymosan (Figure 20A, 20B, 20C, 20D).

#### Pancreatic injury

**Figure 21:**
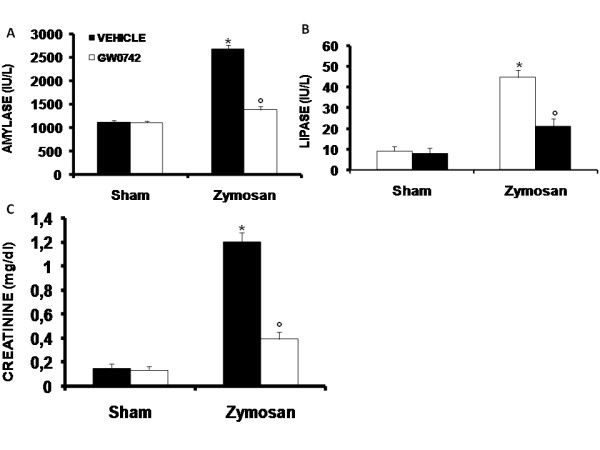
**Effect of GW0742 on pancreatic and renal injury**. Administration of zymosan resulted in significantly increased levels of plasma amylase (**A**), lipase (**B**) and creatinine (**C**). GW0742 treatment significantly decreases all these parameters in zymosan treated mice. Data are mean ± standard deviation from n = 10 mice for each group. *P < 0.01 *vs *sham, °P < 0.01 *vs *zymosan + vehicle.

When compared to sham-operated mice, zymosan-administered mice demonstrated significantly high plasma concentrations of lipase (Figure 21A) and amylase (Figure 21B), suggesting the development of pancreatic injury. In contrast, treatment with GW0742 significantly reduced the pancreatic injury caused by zymosan (Figure 21A, 21B).

#### Renal dysfunction

When compared to sham-operated mice, zymosan-administered mice demonstrated significantly high plasma concentrations of creatinine (Figure 21C), suggesting the development of renal dysfunction. In contrast, treatment with GW0742 significantly reduced the renal dysfunction caused by zymosan (Figure 21C).

## Discussion

Little is known about the peroxisome proliferator-activated receptor (PPAR)-β/δ compared to the other members of the steroid hormone nuclear receptor family, PPAR-α and PPAR-γ [30]. Recently, preclinical *in vivo *studies, using high-affinity PPAR-β/δ agonists, have demonstrated efficacy in models of diabetes as well as obesity β-oxidation, suggesting that modulation of the beta/delta isoform may have a role in treating these diseases as well as the metabolic syndrome [30]. Though the mechanism by which PPAR-β/δ acts remains largely unknown and not yet fully characterized, we wanted to demonstrate a possible therapeutic involvement of the PPAR-β/δ isoform in an acute inflammatory disease such as zymosan-induced multiple organ failure. We demonstrated a beneficial role of the PPAR-β/δ agonist, GW0742, as its treatment decreased the development of acute peritonitis, organ dysfunction and injury, which was associated with a severe illness, a survival approximately of 60% and characterized by systemic toxicity, and significant loss of body weight.

Our results demonstrate that GW0742, through the activation of its PPAR-β/δ receptor, not only mediates anti-inflammatory effects but also attenuates cell death and apoptosis processes, ameliorating organ dysfunction and/or improving survival. We clearly demonstrate that GW0742 significantly reduced exudate formation and the degree of PEC count. Moreover, during the study of other inflammatory diseases, it been shown that several transcription factors, important to the regulation of acute inflammation, serve as substrates for PPARs [31,32]. These include the transcription factor NF-κB (Nuclear Factor Kappa-light-chain-enhancer of activated B cells), a protein complex that is found in almost all animal cell types and is involved in cellular responses to stimuli such as stress, cytokines, free radicals, ultraviolet irradiation, oxidized LDL, and bacterial or viral antigens [33-37]. NF-κB plays a key role in regulating the immune response to infection. Consistent with this role, incorrect regulation of NF-κB has been linked to cancer, inflammatory and autoimmune diseases, septic shock, viral infection, and improper immune development [38]. In concurrence with this, multiple organ dysfunction syndrome (MODS) causes liberation of NF-κB p65 by its physiological inhibitor, IκBα, and hence nuclear translocation where it activates the inflammatory pathway. NF-κB p65 function is strikingly altered by PPAR activation. By Western Blot analysis, we report here that zymosan-induced non-septic shock was associated with significant IκB-α degradation as well as increased nuclear localization of NF-kB p65 in the lung at 18 h after zymosan administration. GW0742-treatment significantly reduced IκBα degradation as well as nuclear translocation of p65. Thus, in this *in vivo *model system, GW0742 appeared to inhibit NF-κB activation, maintaining high cytoplasm levels of IkBα.

PPAR-β/δ activation also attenuates the increase of many cytokines, such as TNF-α and IL-1β, involved in the inflammatory response. There is evidence that the pro-inflammatory cytokines, TNF-α and IL-1β help to propagate the extension of a local or systemic inflammatory process [39,40]. In the present study, zymosan-induced shock causes a substantial increase in the levels of both TNF-α and IL-1β in the plasma after 18 h, while it is clear that GW0742 blocks the mechanisms generating an overproduction of TNF-α and IL-1β. These data are confirmed by immunohistochemical localization of these cytokines. Indeed, the assessment of pancreatic, pulmonary and intestinal tissue sections have revealed a higher presence of TNF-α and IL-1β in samples obtained from zymosan-injected mice, while GW0742 treatment exhibited a fall in the immunohistochemical levels. Moreover, the result of high circulating TNF and IL-1β plasma levels is the expression of endothelial adhesion molecules, such as ICAM-1 and P-selectin that play a pivotal role in the rolling and firm attachment of neutrophils to the endothelium [28], regulating the process of neutrophil chemoattraction, adhesion, and emigration from the vasculature to the tissues [41,42]. In this study, we observed that, 18 h after administration, zymosan induced the expression of P-selectin in the endothelium of small vessels and upregulated the surface expression of ICAM-1 and P-Selectin on endothelial cells in the pancreas, lung and gut. In contrast, there was significantly less expression of P-selectin and ICAM-1 in the pancreas, lung and gut obtained from mice treated with GW0742. Accordingly, we found, by assessment of MPO levels, that neutrophil infiltration was significantly reduced upon GW0742 treatment in zymosan-induced injured mice.

Moreover, in zymosan-induced shock and inflammation the role of nitric oxide (NO), a reactive nitrogen species, has been demonstrated [43] because of the induction of iNOS, which contributes to the inflammatory process [24]. NO levels assessed in exudates and plasma, were increased at 18 h after zymosan-injection, while GW0742 decreased the levels of NO. By Western Blot analysis, we have detected the anti-inflammatory action of GW0742 on iNOS expression, which was reduced when compared with zymosan-only injected mice.

Nitrotyrosine formation, along with its detection by immunostaining, was initially proposed as a relatively specific marker for the detection of the endogenous formation "footprint" of peroxynitrite [44] and an increased nitrotyrosine staining is considered as an indication of increased nitrosative stress [45]. Thus, by immunohistochemical localization, we have seen an increase in nitrotyrosine staining in samples of pancreas, lung and gut obtained from zymosan-induced injured mice, while an improvement was due to GW0742 administration.

Therefore, in this experiment, it is not unexpected that we found that multiple organ failure results also in the formation of peroxynitrite and it is well known that the nuclear enzyme poly (ADP-Ribose) synthetase (PARS) activation can be a consequence of peroxynitrite production [46,47]. A novel pathway of inflammation has been proposed in relation to ROS (hydroxyl radical and peroxynitrite) induced strand breaks in DNA, which trigger energy-consuming DNA repair mechanisms and activates PARP, resulting in the depletion of its substrate NAD^+ ^*in vitro *and a reduction in the rate of glycolysis. As NAD^+ ^functions as a cofactor in glycolysis and the tricarboxylic acid cycle, NAD^+ ^depletion leads to a rapid fall in intracellular ATP. This process has been termed '*the PARP Suicide Hypothesis*'. Thus, a markedly immunohistochemical staining of PARP was detected in sections of pancreas, lung and gut from zymosan-treated mice, while, here, we have observed a decrease of PARP activity in samples of mice treated with GW0742.

The processes that lead to the activation of inflammatory mediators, such as NF-kB p65 or TNF-α are also crucially involved and closely associated to apoptotic processes, that occur in FasL expression induced by DNA-damaging agents, such as a genotoxic drug and UV radiation [48]. Fas forms the Death Inducing Signaling Complex (DISC) upon ligand binding, a multi-protein complex formed by members of the *death receptor *family of apoptosis-inducing cellular receptors [49]. In this study, we have clearly shown the degree of cell death, assessed by immunohistochemical localization of FasL and TUNEL staining, which highlights the presence of apoptotic cell bodies. In either case, we found that zymosan-injection causes an increase of FasL expression in tissue sections of pancreas, lung and gut and TUNEL-positive staining with a marked appearance of dark brown apoptotic cells and intercellular apoptotic fragments in pancreas and lung tissues. On the other hand, the GW0742-mediated activation of PPAR-β/δ confirms the beneficial role in multiple organ failure, decreasing the value of previous apoptotic parameters. Apoptosis manifests itself in two major execution programs downstream of the death signal: the caspase pathway and organelle dysfunction, of which mitochondrial dysfunction is the best characterized [50,51]. As the Bcl-2 family members reside upstream of irreversible cellular damage and focus' much of their efforts at the level of the mitochondria, it plays a pivotal role in deciding whether a cell will live or die. The Bcl-2 family of proteins has expanded significantly and includes both pro- as well as anti-apoptotic molecules. Indeed, the ratio between these two subsets helps determine, in part, the susceptibility of cells to a death signal [52].

Thus, the Western Blot analysis on sections of lung tissue to detect Bax and Bcl-2 expression, supported the idea that the zymosan-injection causes an increase of mitochondrial permeability and severe cellular injury, which leads to a higher expression of Bax than Bcl-2 production, whereas GW0742 administration reduced the apoptosis-induced cell death.

Also the immunohistochemical localization of Bax on sections of pancreatic, pulmonary and intestinal tissue has revealed a loss of physiological balance between pro- and anti-apoptotic factors with an increase of Bax and a decrease of Bcl-2 expression in zymosan-administered mice and, in contrast, a reduction of Bax levels in GW0742-treated animals.

To further confirm these data, we found that GW0742 treatment not only prevents lung dysfunction, and reduces zymosan-induced loss of blood PaO_2_, PCO_2_, HCO_3_^- ^and pH levels, but also diminishes other blood parameters, such as the levels of AST, ALT, bilirubin and alkaline phosphatase that are altered after the onset of zymosan-induced MOF. Furthermore, high concentrations of lipase, amylase and creatinine, indicating the degree of MODS, are all reduced by GW0742, as shown here on liver, pancreas, kidney and lung, is significantly attenuated by PPAR-β/δ activation by GW0742, reducing the pathophysiology of MOF. A histological resolution of organ damage to administration of GW0742 was highlighted in pancreas, lung and gut by haematoxylin-eosin staining too. Indeed, the degree of histoarchitectural modifications in these tissues decreased significantly after treatment with GW0742.

## Conclusion

The multiple organ failure, replicated here through zymosan-injection, is a disease with severe implications, involving several mechanisms not yet fully known.

However, our results clearly suggest that the PPAR-β/δ agonist, GW0742 may be used successfully as a therapeutic agent in the treatment of conditions associated with inflammation and multiple organ system dysfunction.

## List of abbreviations

(DMSO): Dimethyl sulfoxide; (PPARs): Peroxisome proliferator-activated receptors; (PMNs): Polymorphonuclear cells; (PAR): poly(ADP-ribose); (MODS): Multiple organ dysfunction syndrome; (MOF): multiple organ failure; (TNF): tumor necrosis factor α; (IL-6): interleukin-6; (ROS): reactive oxygen species; (PARP): poly (ADP-ribose) polymerase; (NO_2_/NO_3_): Nitrite/nitrate; (MPO): Myeloperoxidase activity; (ALT): alanine aminotransferase; (AST): aspartate aminotransferase; (iNOS): inducible nitric oxide.

## Competing interests

The authors declare that they have no competing interests.

## Authors' contributions

MG, CC have carried out the molecular biology studies; RDP. IP, TG have carried out the animal studies; EM has carried out the histological/immunohistochemical studies; EC, AK have drafted the manuscript and performed the statistical analysis. SC, CT, PB have participated in the design of the study, have coordinated the study and have finalized the manuscript. All authors have read and approved the final manuscript.
